# Response of Soil Bacteria to Short-Term Nitrogen Addition in Nutrient-Poor Areas

**DOI:** 10.3390/microorganisms13010056

**Published:** 2025-01-01

**Authors:** Hongbin Yin, Mingyi Xu, Qingyang Huang, Lihong Xie, Fan Yang, Chao Zhang, Gang Sha, Hongjie Cao

**Affiliations:** 1 Institute of Natural Resources and Ecology, Heilongjiang Academy of Science, Harbin 150040, China; yinhbin@163.com (H.Y.); huangqingyang@163.com (Q.H.); xielihong903@163.com (L.X.); yangfan81039@163.com (F.Y.); zhangchao19800106@163.com (C.Z.); shagang_0312@163.com (G.S.); 2 National and Local Joint Laboratory of Wetland and Ecological Conservation, Harbin 150040, China; xumingyi1982@163.com

**Keywords:** nitrogen addition, microbial community structure, diversity, stability, lava plateau

## Abstract

Increasing nitrogen (N) addition induces soil nutrient imbalances and is recognized as a major regulator of soil microbial communities. However, how soil bacterial abundance, diversity, and community composition respond to exogenous N addition in nutrient-poor and generally N-limited regions remains understudied. In this study, we investigated the effects of short-term exogenous N additions on soil bacterial communities using quantitative polymerase chain reaction (PCR) and Illumina Miseq sequencing in an in situ N addition field experiment. The results showed that a low nitrogen addition increased the observed species (Sobs) of the bacterial community, and with the increased nitrogen addition, the Sobs of bacteria gradually decreased, especially the unique OTUs. The relative abundance of *Proteobacteria*, *Actinobacteria*, and *Gemmatimonadetes* increased with increasing nitrogen addition, whereas the relative abundance of *Chloroflexi* and *Firmicutes* decreased. Soil properties play an important role in bacterial community structure at phylum or genus levels. Short-term nitrogen addition increased the proportion of nodes from *Actinobacteria* and *Proteobacteria* in the co-occurrence network and enhanced the stability of the microbial network. *Actinobacteria* may play an important role in constructing the network. Our study aims to explore the effects of nitrogen addition on the diversity, composition, and structure of soil bacterial communities in nutrient-poor areas caused by ecological disturbances.

## 1. Introduction

Soil bacteria are the most abundant [1] and diverse organisms on earth, and account for a large proportion of terrestrial biodiversity [2], with extensive involvement in soil ecological processes, such as organic matter decomposition, nutrient cycling [3], and aggregate formation [4,5]. They also play a key role in regulating terrestrial biogeochemical cycling, plant productivity, and ecosystem functions [2,6,7]. Soil bacterial community composition is influenced by climatic conditions [8], above-ground vegetation type and species composition [9], soil physicochemical properties [10], and soil nutrient availability [11]. 

Since the industrial revolution, the average nitrogen deposition rate in China has increased from 13.2 kg ha^–2^ a^–1^ in the 1980s to 21.1 kg hm^–2^ a^–1^ in the early 21st century [12] and is higher than the global average value due to the dramatic increase in nitrogen released into the environment from anthropogenic activities, such as agricultural fertilization and fossil fuel combustion [13,14]. Excessive nitrogen inputs may lead to changes in bacterial communities [15]. Global nitrogen deposition has become one of the most important drivers of microbial diversity loss in terrestrial ecosystems [16]. In the past decades, the effects of nitrogen deposition on soil bacterial community diversity were extensively studied in various ecosystems [17] and meta-analyses [18,19]. The findings suggest that nitrogen deposition may lead to the extinction of bacterial species adapted to nutrient-deficient soils by altering soil nutrient conditions (e.g., soil N availability and acidification) [20,21] and plant community composition, diversity, etc. [17,22], which in turn affects bacterial community structure, diversity, and function [21,23]. 

However, recent studies have shown that microbial diversity responds inconsistently to N addition. Some studies have shown that high levels of N addition significantly reduced bacterial diversity and the bacterial community structure [18,19,22]. Soil pH was identified as a key factor regulating soil microbial community structure and diversity [24]. Meanwhile, a number of studies have found that nitrogen-induced soil acidification leads to the loss of bacterial diversity [25,26]. In addition, nitrogen addition changes nutrient uptake and the metabolic activity of plant roots, affects rhizosphere micro-environments, and leads to changes in microbial diversity and structures [27,28]. At present, there are inconsistent results of N input effects on bacterial community diversity [29]. This indicates that the relationship between bacterial community diversity and nitrogen input may be non-linear [30]. Microbial communities are large, and the interactions of microorganisms are complex [31,32]. When the nitrogen input reaches a critical level, the balance of the relationship between microbial taxa will be broken, which will lead to some species’ extinction [33,34] thus reducing the diversity of microbial communities.

On the other hand, high N inputs may also alter species interactions [35], and interactions between microbial species may shift from mutually beneficial symbiosis to competition [36]. Thus, considering mutualistic symbiosis in promoting diversity [37], a shift from mutualistic symbiosis to competition may lead to a dramatic decrease in diversity after high levels of N input and altered soil nutrient limitation, resulting in communities with low diversity [38]. Although many studies have focused on the response of bacterial communities to high nitrogen inputs, there is still limited understanding on how nitrogen inputs affect bacterial diversity and whether a threshold exists along nitrogen addition gradients, particularly in nutrient-poor regions.

The Wudalianchi volcanic group is a well preserved monogenetic volcano group in China. The most recent eruption occurred in 1719–1721, during which a large number of high potassium basaltic lava flows effused, covering the original soils with outpouring lava. The newly formed lava terraces were nutrient-poor, providing a completely new and extreme habitat for microorganisms. Microorganisms, including bacteria, fungi, and other microbes, colonized the rock surface [39]. Through their metabolic activities, microorganisms contribute to the weathering and mineral decomposition of lava, accelerate the formation and development of soils, and facilitate the process of nutrient cycling such as carbon, nitrogen, and phosphorus [40], creating the appropriate conditions for soil ecosystem development [41]. Despite the fact that microorganisms colonies on volcanic rocks are essential for ecosystem establishment and development, there is still a lack of adequate knowledge on how these microorganisms respond to nitrogen deposition. The aim of this study was to investigate the effects of exogenous nitrogen addition on the composition and diversity of bacterial communities in nutrient-poor soil, and to reveal key factors influencing bacterial community structures. The cause-and-effect relationship between soil biodiversity and the stability of microbial structures needs to be investigated from many angles [42]. In a region with limited nutrients, such as volcanic soil, soil nutrient status does enhance microbial diversity and the stability of the microbial structure [42,43]. However, exogenous nitrogen input may improve soil nutrient status and increase soil microbial community diversity in a short time. At the same time, exogenous nitrogen input may break the fragile balance of nutrient stoichiometry in nutrient-limited regions. Therefore, the response of microbial diversity to nitrogen input may be non-linear. We hypothesized that when the nitrogen input reaches a certain level, the soil microbial diversity will reach a threshold value, beyond which the microbial diversity will rapidly decline.

## 2. Materials and Methods

### 2.1. Site Description 

**Figure 1:**
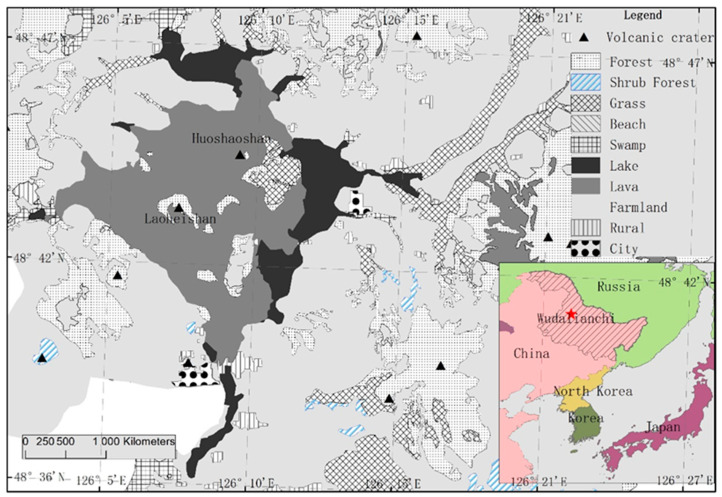
A map of the study area and its overview.

The study site is located in Wudalianchi World Geopark (126°00′~126°45′, 48°30′~48°50′), which is situated in the southwest of Heihe City, Heilongjiang Province, in the transition zone between the southwest foothills of the Xiaoxinganling Mountains and the Songnen Plain (Figure 1). The study site belongs to a temperate continental monsoon climate, with long and cold winters and short and cool summers. The annual average temperature was −0.5 ℃, the annual average frost-free period was about 121 days, and the annual average rainfall was 476.33 mm. The experiment was carried out on the lava terrace of Laoheishan, which erupted only 300 years ago. Due to the short time of lava terrace formation and the slow weathering rate of the lava, soil layers were not formed obviously, and the surface is dominated by large blocks of lava, volcanic gravels, cinder, and ash, with the distribution of higher and lower plants [44]. The soil nutrients in the experimental area are poor [45]. The initial soil properties were as follows: pH(H_2_O) 6.67±0.05, total carbon 6.24 ± 0.18 g/kg, total nitrogen 0.41 ± 0.05 g/kg, ammonia nitrogen (NH_4_^+^-N) 1.97 ± 0.20 mg/kg, nitrate nitrogen (NO_3_^—^N) 0.77 ± 0.08 mg/kg, and available phosphorus (AP) 1.56 ± 0.15 mg/kg. 

### 2.2. Experimental Design, Sample Collection, and Processing

Fifteen experimental plots were established on the terrace with relatively uniform environmental conditions growing dwarf curvilinear *Populus koreana* communities. The field experiment included five different N treatments: 0 (CK), 2 g N m^−2^ yr^−1^ (N1), 4 g N m^−2^ yr^−1^ (N2), 8 g N m^−2^ yr^−1^ (N3), and 16 g N m^−2^ yr^−1^ (N4). We used ammonium chloride as the N fertilizer. Nitrogen fertilization experiments were conducted four times from May to August in 2022–2023, and the nitrogen fertilizer was dissolved in 10 L of deionized water, which was then completely dissolved and transferred to the spraying containers, and uniformly sprayed onto the surface of each plant community. The CK treatment was sprayed with equal amounts of deionized water, and the same amount of nitrogen fertilizer was applied each time for 2 years.

Each fertilization treatment had three replicated plots, with an area of 10 m × 10 m for each plot. The distance between two experiment plots was more than 10 m in order to avoid the interference of fertilization. Soil samples were collected 7 days after fertilization. From each replicate plot, sixteen soil cores (0–10 cm) were collected and pooled to minimize within-plot variation. After the soil was pooled in sterile plastic bags, they were taken back to the laboratory on ice. All the samples were sieved (2 mm), thoroughly homogenized, and divided into two parts: one part was air-dried for soil analysis; the other part was stored at −80℃ for DNA extraction and subsequent molecular analysis. 

### 2.3. Soil Properties Analysis

Soil pH was measured with a pH meter (Sartorius/PB-10, Gottingen, Germany) using a 1:2.5 (*w*/*v*) soil to water ratio. Soil moisture content (moisture) was measured gravimetrically by oven drying the fresh soil samples to a constant weight at 105 ℃ (Taisite/WGL-625B, China). Soil total organic carbon (TOC) and total nitrogen (TN) were determined by the elemental analyzer method (Arvato/EA3000, Italy). Nitrate nitrogen (NO_3_^−^-N) and ammonium nitrogen (NH_4_^+^-N) were extracted by 2 mol L^−1^ KCl and measured using a continuous flow analyzer (SKALAR/SAN^++^, The Netherlands). Available phosphorus (AP) was determined by the Mo-Sb anti-spectrophotometry method after being extracted with 0.5 mol L^−1^ NaHCO_3_. Total phosphorus (TP) and available phosphorus (AP) were determined by the Mo-Sb anti-spectrophotometry method after being digested with H_2_SO_4_-HClO_4_ and extracted with 0.5 mol L^−1^ NaHCO_3_, respectively (Thermo Fisher/Evolution 300, Waltham, MA, USA). Total potassium (TK) and available potassium (AK) were determined by sodium hydroxide fusion-flame photometry and 1 mol L^−1^ of NH_4_OAC solution leaching-flame photometry, respectively (Yuefeng/FP6410, Shanghai, China). Dissolved organic carbon (DOC) and dissolved organic nitrogen (DON) were extracted with distilled water and determined by Multi N-C 2100 (Jena/multi C/N 2100S, Jena, Germany).

### 2.4. DNA Extraction, Quantitative Real-Time PCR (qPCR), PCR Amplification, and Illumina Sequencing

DNA was extracted from 0.5 g of soil using FastDNA^®^ SPIN Kit for Soil (Carlsbad, CA, USA) according to the manufacturer’s instructions. The concentration and purity of the DNA were determined using NanoDrop 2000 UV-vis spectrophotometer (Thermo Fisher Scientific, Waltham, MA, USA), and DNA quality was determined by 1% agarose gel electrophoresis. The V3-V4 hypervariable region of the 16S rRNA gene was amplified using the primers 338F (5′-ACTCCTACGGGGAGG CAGCAG-3′) and 806R (5′-GGACTACVSGGGTATCTAAT-3′). The PCR mixture included 4 μL 5×FastPfu Buffer, 2 μL 2.5 mM dNTPs, 0.8 μL each primer (5 μM), 0.4 μL FastPfu Polymerase, 10 ng of Template DNA, and ddH2O to a final volume of 20 μL. PCR amplification cycling conditions were as follows: initial denaturation at 95 ℃ for 3 min, followed by 27 cycles of denaturing at 95 ℃ for 30 s, annealing at 55 ℃ for 30 s and extension at 72 ℃ for 45 s, and single extension at 72 ℃ for 10 min, ending at 4 ℃. The PCR product was extracted from 2% agarose gel and purified using the AxyPrep DNA Gel Extraction Kit (Promega, Beijing, China) according to the manufacturer’s instructions, and quantified using Qubit 4.0 (Thermo Fisher Scientific, MA, USA). Purified amplicons were pooled in equimolar and paired-end sequenced on the Illumina MiSeq platform by Majorbio Bio-Pharm Technology Co., Ltd. (Shanghai, China). 

The gene copy number of the bacterial 16S rRNA was determined using qPCR with a GeneAmp® 9700 PCR instrument (ABI, San Diego, CA, USA). The same primers employed for the sequencing approach were used to analyze the 16S sequences. The reaction mixture contained 2 μL of the template DNA, 10 μL of SYBR Green Ⅰ PCR master mix (Applied Biosystems, Waltham, MA, USA), 0.2 μL of each primer (10 μmol L^−1^), and was filled with sterile deionized water to 20 μL. The qPCR conditions involved pre-denaturation at 95 ℃ for 3 min, followed by 40 cycles of denaturation at 95 ℃ for 45 s, and annealing at 55 ℃ for 30 s. Standard curves were obtained using tenfold serial dilutions of plasmid DNA. All of the amplifications were run in triplicate.

### 2.5. Data Processes

Raw sequences were demultiplexed, quality-filtered by fastp version 0.20.0 [46], and merged using FLASH version 1.2.7 (San Fran). The processed sequences were assigned to operational taxonomic units (OTUs) defined by 97% similarity cutoff using UPARSE (version 7.1, http://drive5.com/uparse/, accessed on 1 January 2024), and chimeric sequences were identified and removed using UCHIME. Bacteria were classified by the SILVA reference database (version 119, http://www.arb-silva.de, accessed on 1 January 2024) with the Ribosomal Database Project (RDP) Classifier algorithm.

### 2.6. Statistical Analysis

Statistical analyses were conducted using SPSS 25.0 (IBM Corporation, Armonk, NY, USA) and R software (version 4.3.1). Alpha diversity indices, including species observed (Sobs), Chao1, Shannon, and PD diversity were calculated using vegan packages based on OTU information. One-way ANOVA and Duncan’s test were used to compare the means of soil properties, soil bacterial α-diversity, and the relative abundance of the dominant phyla and genera. Bacterial community compositions were ordinated using principal coordinate analysis (PCoA) based on the Bray–Curtis dissimilarity matrices in the vegan package. The effects of N addition on soil bacterial communities were assessed by Aniosm with 999 permutations. A redundancy analysis (RDA) was conducted to elucidate the relationships between the soil bacterial community and soil properties in different nitrogen addition treatments (Table S1). Pearson’s correlation was used to assess the relations between soil properties and bacterial α-diversity indices, and the relative abundance of the dominant phyla and genera. Threshold indicator species analyses (TITAN) was used to identify the indicator species and their threshold value in the “TITAN2” package. TITAN is a non-parametric statistical approach used to detect change points in microbial taxa distribution along an environmental stressor. Two types of indicator species including positive (z+) indicator OTUs and (z−) indicator OTUs were defined as OTUs that increase and decrease in relative abundance along with stress gradients, respectively.

To examine the relationship and interactions between different microbial species, co-occurrence networks based on Spearman’s rank were performed using abundance data of OTU levels of bacterial communities. The co-occurrence patterns were explored based on strong (Spearman’s correlations >|0.9|) and significant correlations (*p* < 0.01). *p*-values were adjusted for multiple testing using the false discovery rate (FDR) according to Benjamini controlling procedure [47]. Gephi (version 0.9.2, http://gephi.org/, accessed on 1 January 2024) was used to visualize network graphs with a Fruchterman–Reingold layout. To further describe the topological properties of networks, several indicators, including the number of nodes and edges, average degree, path length, diameter, clustering coefficient, density, and modularity, were computed using the ‘igraph’ package in R. The correlation network was visualized using Gephi software. Based on within-module connectivity (Zi) and among-module connectivity (Pi), nodes were categorized into four types: (1) network hubs (highly connected within the entire network, Zi > 2.5, Pi > 0.62); (2) module hubs (highly connected within a module, Zi > 2.5, Pi < 0.62); (3) connectors (link modules, Zi < 2.5, Pi > 0.62); and (4) peripherals (less connectivity with other species, Zi < 2.5, Pi < 0.62) [48]. Keystone species were identified using Zi and Pi values. Network hubs, module hubs, and connectors play crucial roles in network topology, and are thus termed keystone species.

## 3. Results

### 3.1. Soil Bacterial Abundance and Diversity Characteristics of Bacteria in Different Nitrogen Addition Treatments

Bacterial 16S rRNA gene abundance varied from 7.18 × 10^9^ to 2.42 × 10^10^ copies g^−1^ soil. Nitrogen addition significantly increased the abundance of bacteria (*p* < 0.05) compared to CK. The abundance of the N1 treatment was higher than other treatments (Figure S2).

Our experiment resulted in a total of 33,587 high-quality sequences of bacterial communities ranging from 41,442 to 49,199 reads per sample. The sequences were binned into 24,111 bacterial OTUs with a 97% identity threshold. For the downstream analysis of microbial sequences, bacterial datasets were rarefied to 33,117 sequences.

Venn analysis (Figure S1) showed that the number of identified bacterial OTUs in CK, N1, N2, N3, and N4 was 9693, 12154, 11366, 9685, and 8699, respectively. A total of 2850 shared OTUs were present in all treatments, and soils from CK, N1, N2, N3, and N4 had 2377, 2941, 2297, 1262, and 883 unique OTUs, respectively. 

Sobs, Shannon, Chao1, and PD were generally significantly different under different nitrogen addition rates (Table 1). Sobs, Shannon, and PD in the N1 treatment were significantly higher than those in CK, and they decreased with nitrogen addition. Chao1 in the CK was significantly lower than that in other nitrogen addition treatments but increasing nitrogen addition did not affect Chao1 significantly.

### 3.2. Bacterial Community Composition in Different Nitrogen Addition Treatments

**Figure 2:**
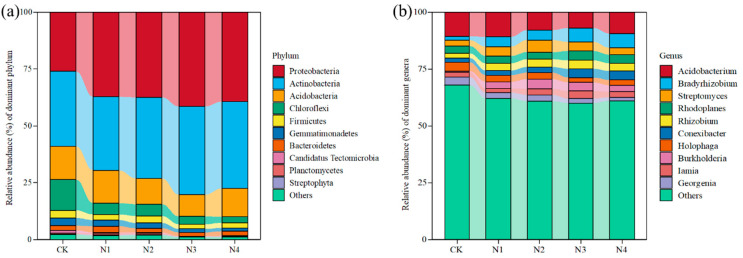
Composition of bacteria of different nitrogen addition treatments on phyla level (**a**) and genera level (**b**).

At the phylum level, the dominant bacterial phyla consisted of *Proteobacteria*, *Actinobacteria*, *Acidobacteria*, *Chloroflexi*, *Firmicutes*, and *Gemmatimonadetes* (Figure 2a). Although the dominant phyla of the bacteria in all soils were consistent, changes in the relative abundance of the dominant taxa were observed across different treatments (Figure S3). There was a higher abundance of *Proteobacteria* and a lower abundance of *Chloroflexi*, *Firmicutes*, and *Gemmatimonadetes* in N addition treatments compared to CK (*p* < 0.05) (Figure S3).

At the genus level, the dominant bacterial genera were *Acidobacterium*, *Bradyrhizobium*, *streptomyces*, *Rhodoplanes*, *Rhizobium*, *Conexibacter*, *Holophaga*, *Burkholderia*, *Iamia*, and *Georgenia* (Figure 2b). Nitrogen addition significantly increased the relative abundance of *Streptomyces*, *Conexibacter*, *Bradyrhizobium*, *Iamia*, and *Burkholderia*, but decreased the relative abundance of *Holophaga*, *Georgenia*, and *Acidobacterium* (*p* < 0.05) (Figure S4).

### 3.3. Differences in Bacterial Community Composition and Its Drivers Across Nitrogen Addition Treatments

**Figure 3:**
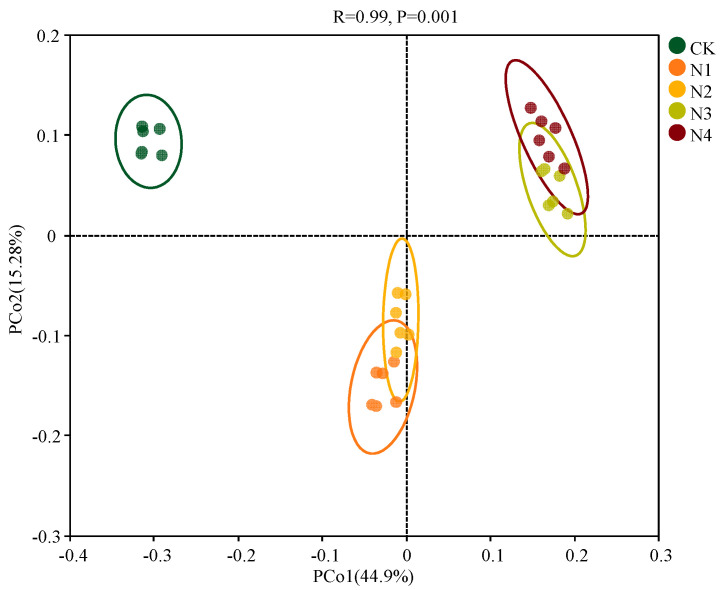
PCoA of bacterial communtiies at different nitrogen addition treatment on OTUs.

PCoA and Anosim analysis showed a clear distinction in bacterial community compositions of the N addition treatments. The first and second principal coordinates explained 44.9% and 15.28, respectively, of the differences among the treatments (Figure 3). The bacterial communities were distributed into three groups: CK, N1-N2, and N3-N4. The distribution of OTUs was influenced by N addition. 

**Figure 4:**
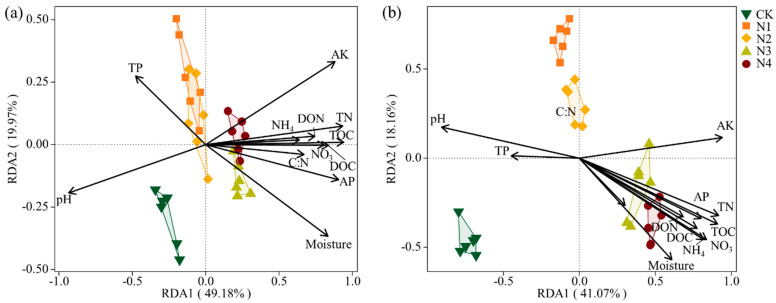
Redundancy analysis (RDA) of relationship between soil properties and bacterial community structures at phylum level (**a**)and genus level (**b**)**.**

RDA was conducted to reveal the impacts of environmental factors on bacterial phyla and genera (Figure 4). At the phylum level, the first two axes of the RDA accounted for 69.08% of the total variation in the bacterial community. At the genus level, the first axis of the RDA explained 41.07% of the variation in the genus community, and the second axis explained 18.16% of the variation. It is worth noting that, among all the environmental factors, AK, TOC, pH, and TN were the most important drivers shaping the bacterial communities (*p* < 0.001). 

Pearson correlation analyses were conducted to further characterize the relationships between bacterial diversity, dominant phyla, dominant genera, and environmental factors (Figure S5). Moisture, DON, and NH_3_^- -^N were significantly negatively correlated with Shannon and PD_faith indices (*p* < 0.05). Moisture and NH_3_^- -^N were significantly negatively correlated with Sobs. pH was significantly negatively correlated with Chao1. The Pearson correlations between the dominant phyla and environmental factors showed that *Proteobacteria* and *Actinobacteria* were significantly positively correlated with most environmental factors and significantly negatively correlated with pH. On the contrary, *Chloroflexi*, *Fimicutes*, and *Gemmatimonadetes* were significantly positively correlated with pH and significantly negatively correlated with most of the other environmental factors. *Acidobacterium* and *Burkholderia* showed no significant correlation with environmental factors, except that *Acidobacterium* had a significantly positively correlation with AP. Other dominant genera showed significant correlations with most of the environmental factors (Figure S7).

### 3.4. Threshold Indicator Taxa Analysis of Bacterial Communities in Different Nitrogen Addition Treatments

**Figure 5:**
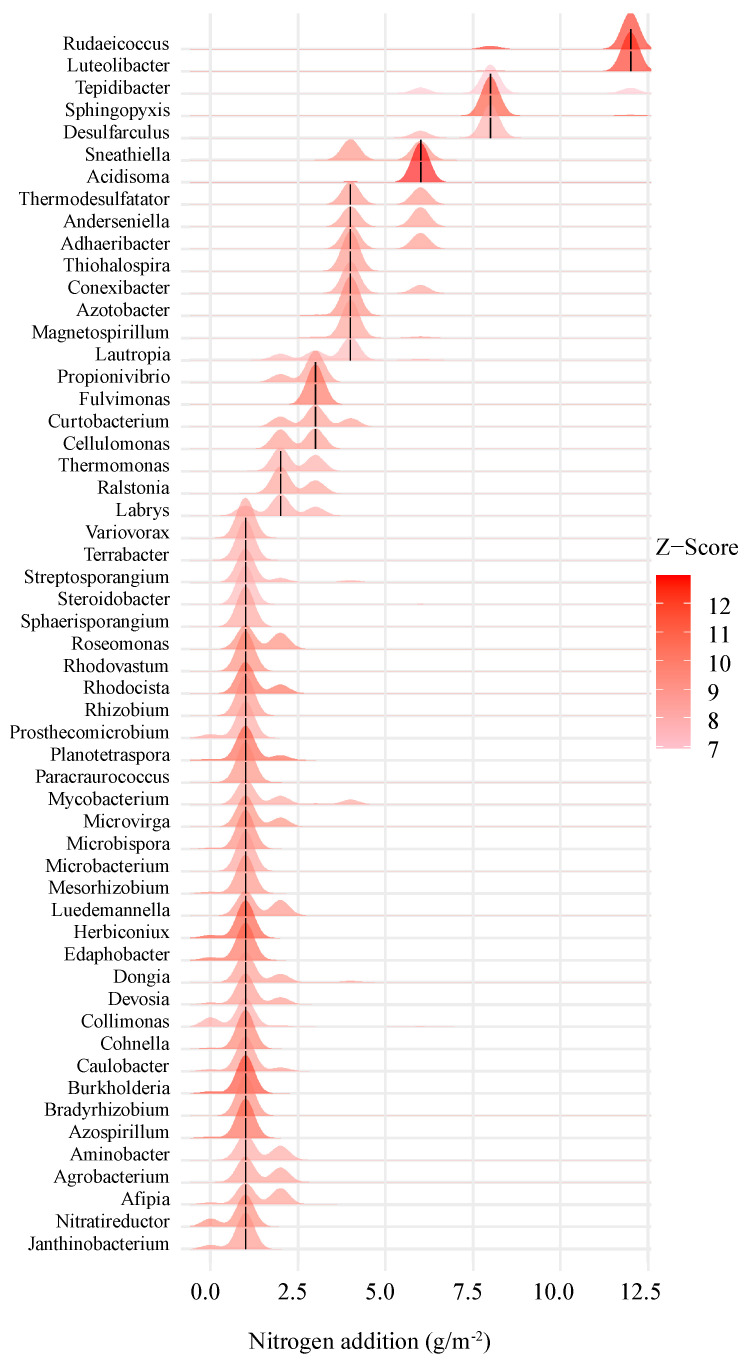
The taxonomy analysis of the threshold indicators (TITAN) showed that the relative abundance of the genera increased with the amount of nitrogen addition. In the figure, the positions of the black short lines in the red peak patterns are the change points observed by the index taxa of the screening criteria. The red peak patterns intersecting the black short lines are the 5th and 95th quantiles of the bootstrap distribution of the change points for each taxon, i.e., confidence or variability bands such as those illustrated in Figure 5. In the figure, the color depth of the peak pattern is proportional to the indicator *z* value, so darker peak patterns are taxa with stronger relative responses to the nitrogen gradient.

**Figure 6:**
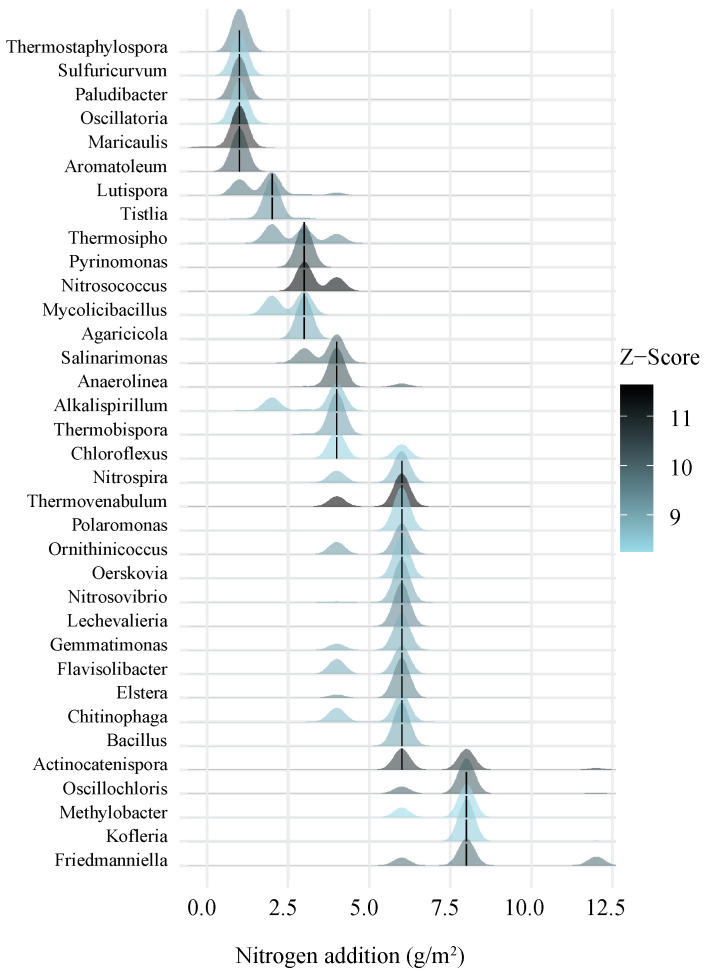
The taxonomy analysis of the threshold indicators (TITAN) showed that the relative abundance of the genera decreased with the amount of nitrogen addition. In the figure, the positions of the black short lines in the blue peak patterns are the change points observed by the index taxa of the screening criteria. The blue peak patterns intersecting the black short lines are the 5th and 95th quantiles of the bootstrap distribution of the change points for each taxon, i.e., confidence or variability bands such as those illustrated in the figure. In the figure, the color depth of the peak pattern is proportional to the indicator *z* value, so darker peak patterns are taxa with stronger relative responses to the nitrogen gradient.

We investigated the turnover of indicator genera using TITAN, which is based on increases (z+) or decreases (z-) in the abundances of indicator genera (Figure 5 and Figure 6) among the bacterial communities. The indicator genera that belonged to Proteobacteria, Actinobacteria, Acidobacteria, Firmicutes, Bacteroidetes, Verrucomicrobia, and Themodesulfobacteria were z+ as the nitrogen addition rates increased, and the indicator genera that belonged to Proteobacteria, Actinobacteria, Acidobacteria, Firmicutes, Bacteroidetes, Cyanobacteria, Nitrospirae, and Gemmatimonadetes were z- as the nitrogen addition rates increased. These results indicated that the nitrogen addition rate affected the compositions of the bacterial communities.

### 3.5. Network Analysis of Bacterial Communities in Different Nitrogen Addition Treatments

**Figure 7:**
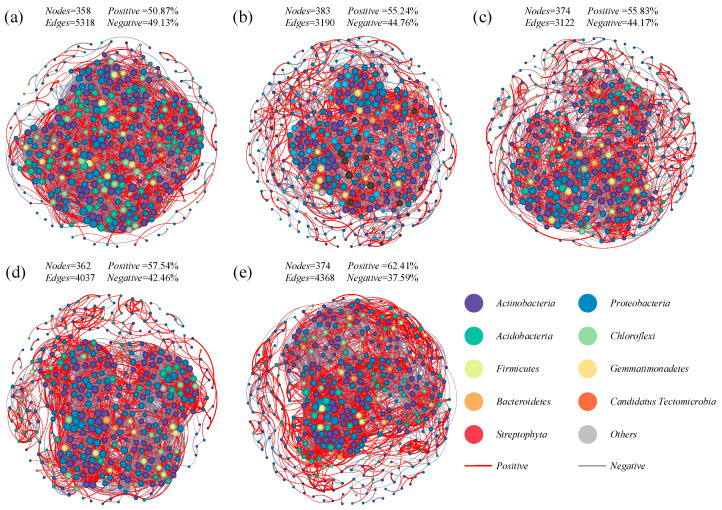
The co-occurrence networks and topological properties of the soil microorganisms in CK (**a**), N1 (**b**), N2 (**c**), N3 (**d**) and N4 (**e**), respectively. The size of each node was proportional to the number of degrees. The classification levels of the microorganisms in the co-occurrence network was phylum. The phyla with an average abundance of less than 1% were classified as “others”.

Based on the significant and strong correlations (ρ > 0.90, *p* < 0.01), we assessed the bacterial co-occurrence networks for each N addition treatment seperately (Figure 7), and the topological properties involved are list in Table S2. Nitrogen addition strongly affected the bacterial co-occurrence pattern. Compared to CK, the number of total edges in N1, N2, N3, and N4 decreased by 40.01%, 41.29%, 24.09%, and 17.86%, respectively; the network densities in N1, N2, N3, and N4 decreased by 46.99%, 45.78%, 25.30%, and 24.10%, respectively; the average degrees in N1, N2, N3, and N4 decreased by 43.93%, 43.80%, 24.93%, and 21.38%, respectively; and the average path length in N1, N2, N3, and N4 increased by 48.47%, 42.27%, 35.33%, and 29.97%, respectively. The total edges, network densities, and average degrees of the medium and high nitrogen addition treatments (N3 and N4) were higher that those of the low nitrogen addition treatments. The average path length showed the opposite trend. Generally, these results indicated that nitrogen addition tended to produce a less complex and connective network compared to CK. The co-occurrence networks of the medium high nitrogen addition rates (N3 and N4) were more complex than those of the low nitrogen addition rate; nitrogen addition decreased the connectivity of the co-occurrence network. 

**Figure 8:**
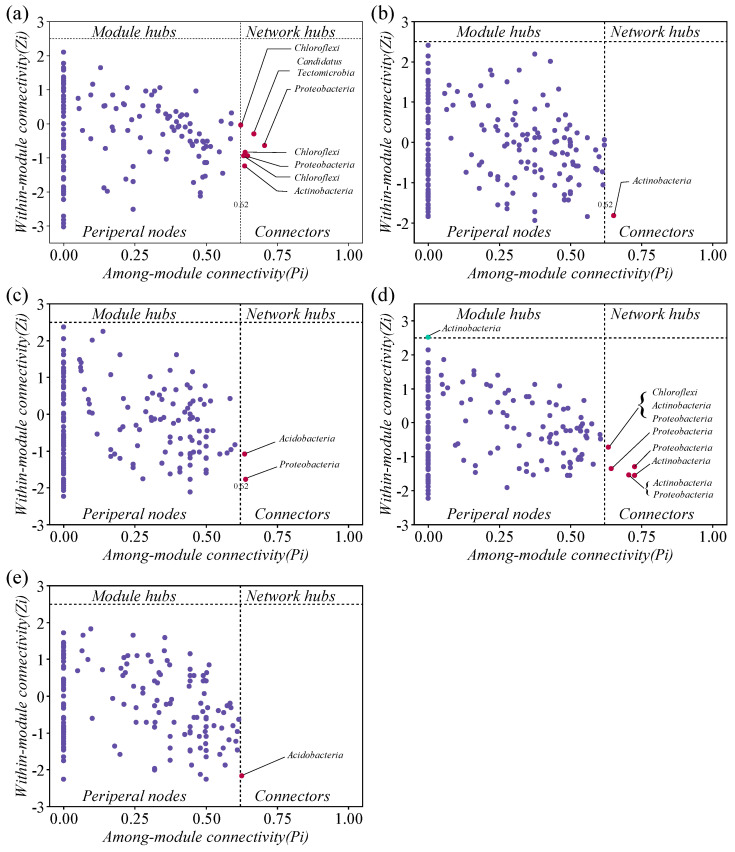
A *Zi-Pi* plot showing the distribution of OTUs based on their topological roles in the bacteria networks under CK (**a**), N1 (**b**), N2 (**c**), N3 (**d**) and N4 (**e**), respectively. The threshold values of *Zi* and *Pi* for categorizing the OTUs were 2.5 and 0.62, respectively.

The intertmodular connectivity (*Pi*) and intramodular connectivity (*Zi*) of the bacterial molecular ecological network in each nitrogen addition treatment indicated that most nodes of the soil bacteria were peripheral (*Zi* < 2.5, *Pi* < 0.62) (Figure 8), occupying a low position in the bacterial community. All OTUs with *Zi* ≥ 2.5 and *Pi* ≥ 0.62 were identified as keystone species. The bacterial networks hosted some keystone species belonging to the phyla *Proteobacteria* (7 OTUs), *Actinobacteria* (5 OTUs), *Chloroflexi* (4 OTUs), *Acidobacteria* (2 OTUs), and *Candidatus Tectomicrobia* (1 OTUs).

## 4. Discussion

Biogeochemical processes and ecosystem functioning directly relate to the abundance, diversity, and composition of soil microbial communities [49]. It is believed that plants provide C through photosynthesis to soil microbes in exchange for nutrients, such as N [50]. However, when the soil availability of N increases, plants could release less C to soil microorganisms and fail to obtain sufficient N [51]. Thus, as N input increases, plants allocate less C belowground, altering the nutrient condition of belowground ecosystems [52] that could significantly affect the abundance, composition, and diversity of soil microbial communities [26,53].

### 4.1. Effect of Nitrogen Addition on Bacterial Diversity

In this study, the low nitrogen addition treatments (N1 and N2) increased bacterial Sobs and alpha diversity, whereas N3 and N4 decreased the alpha diversity of the soil bacterial community with the increasing nitrogen addition rate (Table 1), suggesting that the bacterial diversity response to N addition was non-linear, which is consistent with Liu et al.’s findings [54]. Meanwhile, in nutrient-limited ecosystems, N addition alleviated N limitation of microorganisms and increased the diversity of bacterial communities [55], but excessive N addition could reduce the soil microbial biomass and diversity [22]. Zhang et al. (2022) also showed that low N addition (40 kg N ha^−1^ yr^−1^ ) increased the diversity of soil bacterial communities in a wetland in Northeastern China [56]. A meta-analysis conducted by Wang et al. [57] also showed that the bacterial richness index (Chao1) increased significantly at a N addition rate of 0-5 m^−2^ yr^−1^, and the alpha diversity of the soil bacteria decreased with increasing N additions rates (0–5, 5–10, 10–20, and >20 g N m^−2^ yr^−1^). The increase in bacterial alpha diversity with low nitrogen addition can be explained by the alleviation of nitrogen limitation [58]. Wei et al. [59] and Zeng et al. [60] showed that high nitrogen (100 kg N ha^−1^ yr^−1^ and 240 kg N ha^−1^ yr^−1^) additions inhibit or even decrease the diversity of soil bacteria. 

The TITAN analysis indicated that the threshold for N-induced bacterial community structure changes was between 4 and 8 g N m^−2^ yr^−1^. As is consistent with our hypothesis, we found that bacterial diversity was not affected by low levels of N addition, but sharply declined between 4 and 8 g N m^−2^ yr^−1^. This result implies that bacterial diversity responds non-linearly rather than linearly to N addition, which is in line with the results of Liu et al. [54]. At the same time, under the low nitrogen addition treatments (N1 and N2), the number (40) of bacterial genera with increased relative abundance was greater than those (13) with decreased relative abundance. On the contrary, under the condition of N3 and N4, the number (22) of bacterial genera with reduced relative abundance was greater than those (15) with increased relative abundance. Rare bacteria (relative abundance < 0.01%) accounted for 62.64% of the bacteria indicator genera. These results further explained the variation in the observed bacterial numbers and diversity, suggesting that rare bacteria may play important roles in the effects of nitrogen addition on the diversity of soil bacterial communities.

Studies have shown that nitrogen addition may increase nitrogen and phosphorus content in plant tissues [61] provide high-quality litters to the soil [62,63], and that the abundant resources form new ecological niches for microbial colonization, which will have a positive effect on the diversity of bacterial communities considering the interaction of microbial communities. In this case, the increase in soil available nitrogen prompted bacteria to shift from oligotrophic strategies adapting to poor conditions to copiotrophic strategies adapting to rich conditions [64,65]. It was shown that as N availability increases and nitrification enhances, soil pH decreases and leads to soil acidification. Soil acidification could cause bacterial diversity decreasing, shown by trends of bacterial diversity and soil pH [60,66]. In this study, Shannon, Sobs, and phylogenetic diversity showed similar trends with soil pH, but the relationships were not significant. The result was consistent with a large-scale meta-analysis of forest and grassland ecosystems [57], which is contrary to a study that conducted long-term N-addition experiments in forest ecosystems [67]. This suggests that the effect of nitrogen addition on the diversity of soil bacterial communities relates to ecosystem type, study location, type of fertilizer, and a balance between the facilitating effect of increased nitrogen availability and the inhibiting effect of soil pH.

### 4.2. Effect of Nitrogen Addition on Bacterial Species’ Compositions

Studies have shown that the response of soil microbial communities to N addition is influenced by the N addition rate [68] and N addition duration [69,70]. The relative abundance of *Proteobacteria* typically increases with N addition [26], and the relative abundance of *Firmicutes* decreases with N addition [71]. However, He et al. [69] showed that short-term nitrogen addition had no significant effect on soil bacterial communities. In this study, a 2-year short-term nitrogen addition experiment showed that the relative abundance of *Proteobacteria*, *Actinobacteria*, and *Gemmatimonadetes* gradually increased with nitrogen addition, while the relative abundance of *Chloroflexi* and *Firmicutes* gradually decreased (Figure 2a and Figure S3). This is consistent with most long-term N addition experiments [70,72], but inconsistent with the short-term N addition study conducted by He et al. [68]. The possible reason may be the nutrient-limited condition in the study area, and a short-term exogenous N addition may alleviate the soil nutrient-limited condition effectively, and thus the response of oligotrophic and copiotrophic microbial taxa to N addition was different. Nitrogen input may directly or indirectly induce changes in life history strategies of dominant microbial populations [73]. A study has shown that *Proteobacteria* harbor a wide range of habitats [74], and is considered as a coiotrophic bacterial taxa. Nitrogen addition increases the relative abundance of *Proteobacteria* [75]. At the same time, nitrogen deposition favors bacterial taxa with high lignin modification potential, such as *Actinobacteria* [51,76]. *Acidobacteria* is one of the most abundant and prevalent phyla in soils of various ecosystems [77] and is considered an oligotrophic (K-selective) bacterial taxon [78] and the most common taxa in nutrient-poor soils [2]. Nitrogen addition, improving the soil nutrient condition, may be responsible for decreasing the relative abundance of *Acidobacteria* [60].

Nitrogen deposition can alter a variety of soil properties, affecting microbial community composition by regulating soil N availability and pH [50]. This may directly or indirectly affect the soil microbial community [50]. In this study, the relative abundance of *Proteobacteria* and *Actinobacteria* was significantly negatively correlated with pH (Figure S6). Meanwhile, PCoA based on OTU levels confirmed changes in community composition between the N addition treatments (Figure 3). The first PCoA component separated communities into three clusters based on N addition rates. N1 and N2 treatments clustered together, N3 and N4 treatments clustered together, and the two groups both separated from CK (Figure 3). These results confirm that nitrogen addition has a significant effect on the community structure of soil bacteria [19,51]; 4 g N m^−2^ yr^−1^ may be a threshold for changes in the composition of soil bacterial communities in lava plateaus (Figure 4, Figure 5 and Figure S8). The redundancy analyses (RDA) further illustrated that nitrogen addition significantly changed the bacterial community structure at both the phylum level and genus level (Figure 3). Bacterial community composition was found to be significantly correlated with TN, NO_3_^—^N, NH_4_^+^-N, and DON. Our result is consistent with those of Yan et al. [79] and Fierer et al. [80], who showed that the shift in dominant life history strategies could be a result of the increase in N availability. Soil total nitrogen has a significant influence on microbial community structures [81]. Soil organic carbon was also reported to be the most important factor influencing the bacterial community [82]. Soil pH was reported to decrease with N addition [79,83], and decreased pH led to a change in soil bacterial community [84]. 

### 4.3. Effect of Nitrogen Addition on Stability of Bacterial Communities

Co-occurrence network analysis could provide insights into the changes in bacterial community assembly in soils with different treatments [85]. The advantage of co-occurrence networks is that they reveal the complexity of community connections, as well as key taxa and their responses to changes in soil properties [86,87]. Furthermore, the complexity and stability of microbial networks are significantly reduced under unfavorable environments [88]. In this study, the differentiation in topological features indicates that N addition changed the co-occurrence patterns (Figure 7). It suggests that short-term nitrogen addition enhanced the stability of the microbial network by increasing more linkages between microbial taxa and promoting connections among soil microbial species [17]. Key taxa are drivers of microbial community structures and functions, and their influence is mainly affected by their relative abundance [89]. In addition, key taxa may have more interactions with the community and can serve as indicators of community succession [90]. The study of Chen et al. [91] conducted at the Meijiawu Tea District site suggests that key taxa such as *Firmicutes* and *Actinobacteria* may play an important role in the co-occurrence network and have a relationship with taxa, which are significantly connected. In this study, the percentage of nodes belonging to *Actinobacteria* and *Proteobacteria* in the co-occurrence network of soil bacterial communities with N addition treatments was above 60%. N addition treatments increased the proportion of nodes belonging to *Actinobacteria* and *Proteobacteria* (Figure 7). The increase in dominant taxa may reduce the complexity of soil bacterial communities, which could further lead to the loss of rare taxa. A decrease in unique OTUs with N addition increase can also confirm this finding (Figure 8). *Zi-Pi* analyses revealed that Actinobacteria may play an important role in maintaining bacterial community stability (Figure 8). The possible reason may be that nitrogen addition improved material and energy for microorganisms and increased the relative abundance of *Actinobacteria*, which belongs to copiotrophic taxa. Moreover,, improved environmental conditions promote plant growth and increase the lignin substance of plant litter into the soil, which will further increase the relative abundance of bacterial taxa (e.g., *Actinobacteria*) that have a high potential for lignin decomposition. The key taxa influencing the microbial community structure depends on ecosystem types, nutrient status, and so on. A study conducted on biological soil crust revealed that phylum *Proteobacteria* played a vital role in the bacterial network structure with increasing nutrients contents [92], which might be due to a variety of genera in the phylum *Proteobacteria*-fixing carbon [93]. In nutrient-poor ecosystems, such as desert steppe, soils sampled from a severe desertification stage featured bacteria related to energy source utilization as indicators, including bacteria playing essential roles in oxidizing ammonia to nitrite and fixing carbon dioxide [94], and bacteria adapting to severe environments by using various carbon compounds as growth substrates [95].

## 5. Conclusions

Our results showed that low nitrogen addition (N1 and N2) increased Sobs of the bacterial community, and, with increasing nitrogen addition, the Sobs and unique OTUs both decreased. Nitrogen addition has an effect on the structure of the soil bacterial community, and soil properties drive differential patterns of bacterial communities. Short-term nitrogen addition increased the proportion of nodes occupied by *Actinobacteria* and *Proteobacteria*, which enhanced the stability of the microbial co-occurrence network by increasing more linkages and facilitating connections between soil microbial species. Actinobacteria may play an important role in maintaining community stability. This study was conducted using short-term in situ field experiments. In our future studies, it may be necessary to monitor the effects of continuous exogenous nitrogen addition on soil microorganisms, especially the response of different microbial populations, as well as the interactions between plants and microorganisms, providing insight into predicting the changes in regional environment conditions caused by nitrogen addition.

## Figures and Tables

**Table 1:** The alpha diversity index of bacteria at different nitrogen addition treatments. The data represent mean ± standard error (n = 6). If the letters are the same, the divergence is not significant; on the contrary, divergence is significant at 0.05 levels.

Traetment/Index	CK	N1	N2	N3	N4
Sobs	5467.33 ± 238.89 bc	6676 ± 82.62 a	6259.33 ± 105.67 ab	5688 ± 396.62 bc	5235.33 ± 287.96 c
Chao1	6372.67 ± 254.81 b	7388.82 ± 60.80 a	7457.14 ± 109.95 a	8175.13 ± 479.51 a	7634.54 ± 399.76 a
Shannon	7.42 ± 0.56 bc	7.78 ± 0.03 a	7.61 ± 0.01 ab	7.40 ± 0.14 bc	7.21 ± 0.11 c
PD	251.56 ± 7.38 bc	298.72 ± 4.41 a	275.06 ± 3.28 ab	230.12 ± 21.77 c	217.43 ± 16.08 c

## Data Availability

Data are contained within the article and Supplementary Materials.

## Supplementary Materials

The following supporting information can be downloaded at: https://www.mdpi.com/article/10.3390/microorganisms13010056/s1. Figure S1: A Venn showing the cluster distribution of the bacteria at different nitrogen addition treatments on OTUs level; Figure S2: The variation characteristics of 16S gene copy number at different nitrogen addition treatments; Figure S3: Significance test of bacterial community compositions at different nitrogen addition treatments on phylum level; Figure S4: Significance test of bacterial community compositions at different nitrogen addition treatments on genus level; Figure S5: Heatmap of correlation between bacterial alpha diversity and environmental factors; Figure S6: Heatmap of correlation between nitrogen addition treatments and environmental factors on phylum level; Figure S7: Heatmap of correlation between nitrogen addition treatments and environmental factors on genus level; Figure S8: Bacterial community change point for genus reduced in abundance along with N addition levels showing community threshold at maximum [sum(z)] and 5–95% bootstrap percentile range; Table S1: Redundancy analysis explains importance of variables and tests their significance; Table S2: Topological properties of co-occurrence network obtained from different nitrogen additional treatments.

## References

[1] Liu J., Li S., Yue S., Tian J., Chen H., Jiang H., Siddique K.H., Zhan A., Fang Q., Yu Q. (2021). Soil microbial community and network changes after long-term use of plastic mulch and nitrogen fertilization on semiarid farmland. Geoderma.

[2] Delgado-Baquerizo M., Oliverio A.M., Brewer T.E., Benavent-González A., Eldridge D.J., Bardgett R.D., Maestre F.T., Singh B.K., Fierer N. (2018). A global atlas of the dominant soil bacteria found in soil. Science.

[3] Wei G.S., Li M.C., Shi W.C., Tian R.M., Chang C.Y., Wang Z.R., Wang N.X., Zhao G.X., Gao Z. (2020). Similar drivers but different effects lead to distinct ecological patterns of soil bacterial and archaeal communities. Soil Biol. Biochem..

[4] Crowther T.W., van den Hoogen J., Wan J., Mayes M.A., Keiser A.D., Mo L., Averill C., Maynard D.S. (2019). The global soil community and its influence on biogeochemistry. Science.

[5] Sokol N.W., Slessarev E., Marschmann G.L., Nicolas A., Blazewicz S.J., Brodie E.L., Firestone M.K., Foley M.M., Hestrin R., Hungate B.A. (2022). Life and death in the soil microbiome: How ecological processes influence biogeochemistry. Nat. Rev. Microbiol..

[6] Chen W.J., Zhou H.K., Wu Y., Wang J., Zhao Z.W., Li Y.Z., Qiao L.L., Chen L.L., Liu G.B., Xue S. (2020). Direct and indirect influences of long-term fertilization on microbial carbon and nitrogen cycles in an alpine grassland. Soil Biol. Biochem..

[7] Jansson J.K., Hofmockel K.S. (2020). Soil microbiomes and climate change. Nat. Rev. Microbiol..

[8] Maestre F.T., Delgado-Baquerizo M., Jeffries T.C., Eldridge D.J., Ochoa V., Gozalo B., Quero J.L., García-Gómez M., Gallardo A., Ulrich W. (2015). Increasing aridity reduces soil microbial diversity and abundance in global drylands. Proc. Natl. Acad. Sci. USA.

[9] Prober S.M., Leff J.W., Bates S.T., Borer E.T., Firn E., Harpole W.S., Lind E.M., Seabloom E.W., Adler P.B., Bakker J.D. (2015). Plant diversity predicts beta but not alpha diversity of soil microbes across grasslands worldwide. Ecol. Lett..

[10] Lauber C.L., Hamady M., Knight R., Fierer N. (2009). Pyrosequencing-based assessment of soil pH as a predictor of soil bacterial community structure at the continental scale. Appl. Environ. Microbiol..

[11] Leff J.W., Jones S.E., Prober S.M., Barberán A., Borer E.T., Firn J.L., Harpole W.S., Hobbie S.E., Hofmockel K.S., Knops J.M.H. (2015). Consistent responses of soil microbial communities to elevated nutrient inputs in grasslands across the globe. Proc. Natl. Acad. Sci. USA.

[12] Liu X., Zhang Y., Han W., Tang A., Shen J., Cui Z., Vitousek P.M., Erisman J.W., Goulding K.W., Christie P. (2013). Enhanced nitrogen deposition over China. Nature.

[13] Lekberg Y., Arnillas C.A., Borer E.T., Bullington L.S., Fierer N., Kennedy P.G., Leff J.W., Luis A.D., Seabloom E.W., Henning J.A. (2021). Nitrogen and phosphorus fertilization consistently favor pathogenic over mutualistic fungi in grassland soils. Nat. Commun..

[14] Liu L., Zhang X.Y., Xu W., Liu X.J., Zhang Y., Li Y., Wei J., Lu X.H., Wang S.Q., Zhang W.T. (2020). Fall of oxidized while rise of reduced reactive nitrogen deposition in China. J. Clean. Prod..

[15] Contosta A.R., Frey S.D., Cooper A.B. (2015). Soil microbial communities vary as much over time as with chronic warming and nitrogen additions. Soil Biol. Biochem..

[16] Galloway J.N., Winiwarter W., Leip A., Leach A.M., Bleeker A., Erisman J.W. (2014). Nitrogen footprints: Past, present and future. Environ. Res. Lett..

[17] Liu W.X., Liu L.L., Yang X., Deng M.F., Wang Z., Wang P.D., Yang S., Li P., Peng Z.Y., Yang L. (2021). Long-term nitrogen input alters plant and soil bacterial, but not fungal beta diversity in a semiarid grassland. Glob. Chang. Biol..

[18] Zhou Z.H., Wang C.K., Luo Y.Q. (2020). Meta-analysis of the impacts of global change factors on soil microbial diversity and functionality. Nat. Commun..

[19] Zhou Z.H., Zheng M.H., Xia J.Y., Wang C.K. (2022). Nitrogen addition promotes soil microbial beta diversity and the stochastic assembly. Sci. Total Environ..

[20] Zhou J., Deng Y., Shen L., Wen C., Yan Q., Ning D., Qin Y., Xue K., Wu L., He Z. (2016). Temperature mediates continental-scale diversity of microbes in forest soils. Nat. Commun..

[21] Widdig M., Heintz-Buschart A., Schleuss P.M., Guhr A., Borer E.T., Seabloom E.W., Spohn M. (2020). Effects of nitrogen and phosphorus addition on microbial community composition and element cycling in a grassland soil. Soil Biol. Biochem..

[22] Wang C., Liu D.W., Bai E. (2018). Decreasing soil microbial diversity is associated with decreasing microbial biomass under nitrogen addition. Soil Biol. Biochem..

[23] Wang C., Yang Q., Zhang C., Zhang X., Chen J., Liu K. (2023). Vegetation restoration of abandoned cropland improves soil ecosystem multifunctionality through alleviating nitrogen-limitation in the China Danxia. Front. Plant Sci..

[24] Fierer N. (2017). Embracing the unknown: Disentangling the complexities of the soil microbiome. Nat. Rev. Microbiol..

[25] Ling N., Chen D.M., Guo H., Wei J.X., Bai Y., Shen Q.R., Hu S.J. (2017). Differential responses of soil bacterial communities to long-term N and P inputs in a semi-arid steppe. Geoderma.

[26] Nie Y.X., Wang M.C., Zhang W., Ni Z., Hashidoko Y.S.Y.K., Shen W.J. (2018). Ammonium nitrogen content is a dominant predictor of bacterial community composition in an acidic forest soil with exogenous nitrogen enrichment. Sci. Total Environ..

[27] Wardle D.A., Walker L., Bardgett R.D. (2004). Ecosystem Properties and Forest Decline in Contrasting Long-Term Chronosequences. Science.

[28] Lange M., Eisenhauer N., Sierra C.A., Bessler H., Engels C., Griffiths R.I., Mellado-Vázquez P.G., Malik A.A., Roy J., Scheu S. (2015). Plant diversity increases soil microbial activity and soil carbon storage. Nat. Commun..

[29] Ramirez K.S., Craine J.M., Fierer N. (2012). Consistent effects of nitrogen amendments on soil microbial communities and processes across biomes. Glob. Chang. Biol..

[30] Yao M.J., Rui J.P., Li J.B., Dai Y.M., Bai Y.F., Heděnec P., Wang J.M., Zhang S.H., Pei K.Q., Liu C. (2014). Rate-specific responses of prokaryotic diversity and structure to nitrogen deposition in the *Leymus chinensis* steppe. Soil Biol. Biochem..

[31] Luo J., Liao G., Banerjee S., Gu S., Liang J., Guo X., Zhao H., Liang Y., Li T. (2023). Long-term organic fertilization promotes the resilience of soil multifunctionality driven by bacterial communities. Soil Biol. Biochem..

[32] Bairey E., Kelsic E.D., Kishony R. (2016). High-order species interactions shape ecosystem diversity. Nat. Commun..

[33] Levine H., Jørgensen N., Martino-Andrade A., Mendiola J., Weksler-Derri D., Mindlis I., Pinotti R., Swan S.H. (2017). Temporal trends in sperm count: A systematic review and meta-regression analysis. Hum. Reprod. Update.

[34] Shade A., Peter H., Allison S.D., Baho D.L., Berga M., Bürgmann H., Huber D.H., Langenheder S., Lennon J.T., Martiny J.B. (2012). Fundamentals of Microbial Community Resistance and Resilience. Front. Microbiol..

[35] Callaway R.M., Brooker R.W., Choler P., Kikvidze Z., Lortie C.J., Michalet R., Paolini L., Pugnaire F.I., Newingham B., Aschehoug E.T. (2002). Positive interactions among alpine plants increase with stress. Nature.

[36] Hoek T.A., Axelrod K., Biancalani T., Yurtsev E.A., Liu J., Gore J. (2016). Resource Availability Modulates the Cooperative and Competitive Nature of a Microbial Cross-Feeding Mutualism. PLoS Biol..

[37] Coyte K.Z., Schluter J., Foster K.R. (2015). The ecology of the microbiome: Networks, competition, and stability. Science.

[38] Scheffer M., Carpenter S.R., Lenton T.M., Bascompte J., Brock W.A., Dakos V., van de Koppel J., van de Leemput I.A., Levin S.A., van Nes E.H. (2012). Anticipating Critical Transitions. Science.

[39] Hernández M., Calabi M., Conrad R., Dumont M.G. (2020). Analysis of the microbial communities in soils of different ages following volcanic eruptions. Pedosphere.

[40] Rincón-Molina C.I., Martínez-Romero E., Ruíz-Valdiviezo V.M., Velázquez E., Ruíz-Lau N., Rogel-Hernández M.A., Villalobos-Maldonado J.J., Rincón-Rosales R. (2020). Plant growth-promoting potential of bacteria associated to pioneer plants from an active volcanic site of Chiapas (Mexico). Appl. Soil Ecol..

[41] Garrido-Benavent I., Pérez-Ortega S., Durán J., Ascaso C., Pointing S.B., Rodríguez-Cielos R., Navarro F., de los Ríos A. (2020). Differential Colonization and Succession of Microbial Communities in Rock and Soil Substrates on a Maritime Antarctic Glacier Forefield. Front. Microbiol..

[42] Odum E.P. (1969). The Strategy of Ecosystem Development. Science.

[43] Huang Q.Y., Yang F., Cao H.J., Cheng J.H., Jiang M.Y., Li M.H., Ni H.W., Xie L. (2024). Comparison of Microbial Diversity of Two Typical Volcanic Soils in Wudalianchi, China. Microorganisms.

[44] Zhou Z.Q., Xu L.J., Zhang Y.H., Xia C.M., Li H.G., Liu T. (2011). An analysis of the ecological value of Wudalianchi, Heilongjiang Province, China. Biodiver. Sci..

[45] Cao H.J., Wang L.M., Xu M.Y., Huang Q.Y., Luo C.Y., Xie L.H., Ni H.W. (2019). Soil microbial biomass and enzymes activity under different vegetation types at the new stage volcanic lava platform, Wudalianchi area, northeast China. J. Cent. South Univ..

[46] Chen S.F., Zhou Y.Q., Chen Y.R., Gu J. (2018). fastp: An ultra-fast all-in-one FASTQ preprocessor. Bioinformatics..

[47] Benjamini Y., Krieger A.M., Yekutieli D. (2006). Adaptive linear step-up procedures that control the false discovery rate. Biometrika.

[48] Deng Y., Jiang Y.H., Yang Y.F., He Z.L., Luo F., Zhou J.Z. (2012). Molecular ecological network analyses. BMC Bioinform..

[49] Shi X.Z., Wang J.Q., Müller C., Hu H.-W., He J.Z., Wang J.T., Huang Z.Q. (2020). Dissimilatory nitrate reduction to ammonium dominates soil nitrate retention capacity in subtropical forests. Biol. Fertil. Soils.

[50] Vasar M., Andreson R., Davison J., Jairus T., Moora M., Remm M., Young J.P.W., Zobel M., Öpik M. (2017). Increased sequencing depth does not increase captured diversity of arbuscular mycorrhizal fungi. Mycorrhiza.

[51] Johnson I., Thornley J. (1987). A model of shoot: Root partitioning with optimal growth. Ann. Bot..

[52] Wang Y.S., Cheng S.L., Yu G.R., Fang H.J., Mo J.M., Xu M.J., Gao W.L. (2015). Response of carbon utilization and enzymatic activities to nitrogen deposition in three forests of subtropical China. Can. J. For. Res..

[53] Guan H.L., Zhang Y.Q., Mao Q.G., Zhong B.Q., Chen W.B., Mo J.M., Wang F.M., Lu X.K. (2023). Consistent effects of nitrogen addition on soil microbial communities across three successional stages in tropical forest ecosystems. Catena.

[54] Liu W.X., Jiang L., Yang S., Wang Z., Tian R., Peng Z.Y., Chen Y.L., Zhang X.X., Kuang J.L., Ling N. (2020). Critical transition of soil bacterial diversity and composition triggered by nitrogen enrichment. Ecology.

[55] Han Y., Zhang Z., Wang C., Jiang F., Xia J. (2012). Effects of mowing and nitrogen addition on soil respiration in three patches in an oldfield grassland in Inner Mongolia. J. Plant Ecol..

[56] Zhang R.T., Liu Y.N., Zhong H.X., Chen X.W., Sui X. (2022). Effects of simulated nitrogen deposition on the soil microbial community diversity of a Deyeuxia angustifolia wetland in the Sanjiang Plain. Northeast. China Ann. Microbiol..

[57] Wang X.D., Feng J.G., Ao G., Qin W.K., Han M.G., Shen Y.W., Liu M.L., Chen Y., Zhu B. (2023). Globally nitrogen addition alters soil microbial community structure, but has minor effects on soil microbial diversity and richness. Soil Biol. Biochem..

[58] Averill C., Waring B. (2018). Nitrogen limitation of decomposition and decay: How can it occur?. Glob. Chang. Biol..

[59] Wei H.W., Wang X.G., Li Y.B., Yang J.J., Wang J.F., Lü X.T., Han X.G. (2020). Simulated nitrogen deposition decreases soil microbial diversity in a semiarid grassland, with little mediation of this effect by mowing. Pedobiologia.

[60] Zeng J., Liu X., Song L., Lin X., Zhang H., Shen C., Chu H. (2016). Nitrogen fertilization directly affects soil bacterial diversity and indirectly affects bacterial community composition. Soil Biol. Biochem..

[61] Calvo-Fernández J., Taboada A., Fichtner A., Härdtle W., Calvo L., Marcos E. (2018). Time- and age-related effects of experimentally simulated nitrogen deposition on the functioning of montane heathland ecosystems. Sci. Total Environ..

[62] Blaško R., Forsmark B., Gundale M.J., Lim H., Lundmark T., Nordin A. (2022). The carbon sequestration response of aboveground biomass and soils to nutrient enrichment in boreal forests depends on baseline site productivity. Sci. Total Environ..

[63] Forsmark B., Nordin A., Rosenstock N.P., Wallander H., Gundale M.J. (2021). Anthropogenic nitrogen enrichment increased the efficiency of belowground biomass production in a boreal forest. Soil Biol. Biochem..

[64] Pei Z., Leppert K.N., Eichenberg D., Bruelheide H., Niklaus P.A., Buscot F., Gutknecht J.L.M. (2017). Leaf litter diversity alters microbial activity, microbial abundances, and nutrient cycling in a subtropical forest ecosystem. Biogeochemistry.

[65] Pilkington M.G., Caporn S.J.M., Carroll J.A., Cresswell N., Lee J.A., Reynolds B., Emmett B.A. (2005). Effects of increased deposition of atmospheric nitrogen on an upland moor: Nitrogen budgets and nutrient accumulation. Environ. Pollut..

[66] Yang Y., Cheng H., Gao H., An S.S. (2020). Response and driving factors of soil microbial diversity related to global nitrogen addition. Land Degrad. Dev..

[67] Wang C., Lu X., Mori T., Mao Q., Zhou K., Zhou G., Nie Y., Mo J. (2018). Responses of soil microbial community to continuous experimental nitrogen additions for 13 years in a nitrogen-rich tropical forest. Soil Biol. Biochem..

[68] He W.Y., Zhang M.M., Jin G.Z., Sui X., Zhang T., Song F.Q. (2021). Effects of nitrogen deposition on nitrogen-mineralizing enzyme activity and soil microbial community structure in a Korean pine plantation. Microb. Ecol..

[69] He J.H., Tan X.P., Nie Y.X., Ma L., Liu J.X., Lu X.K., Mo J.M., Leloup J.L., Nunan N., Ye Q. (2023). Distinct responses of abundant and rare soil bacteria to nitrogen addition in tropical forest soils. Microbiol. Spectr..

[70] Xu A.X., Li L.L., Xie J.H., Zhang R.Z., Luo Z.Z., Cai L.Q., Liu C., Wang L.L., Anwar S., Jiang Y.J. (2022). Bacterial diversity and potential functions in response to long-term nitrogen fertilizer on the semiarid loess plateau. Microorganisms.

[71] Zhang K., Ni Y., Liu X., Chu H. (2020). Microbes changed their carbon use strategy to regulate the priming effect in an 11-year nitrogen addition experiment in grassland. Sci. Total Environ..

[72] Ma X., Zhou Z., Chen J., Xu H., Ma S., Dippold M.A., Kuzyakov Y. (2023). Long-term nitrogen and phosphorus fertilization reveals that phosphorus limitation shapes the microbial community composition and functions in tropical montane forest soil. Sci. Total Environ..

[73] Fierer N., Lauber C.L., Ramirez K.S., Zaneveld J., Knight R. (2011). Comparative metagenomic, phylogenetic and physiological analyses of soil microbial communities. ISME J..

[74] Ettema T.J., Andersson S.G. (2009). The alpha-proteobacteria: The Darwin finches of the bacterial world. Biol. Lett..

[75] Jenkins S.N., Rushton S.P., Lanyon C.V., Whiteley A.S., Waite I.S., Brookes P.C., Kemmitt S., Evershed R.P., O’Donnell A.G. (2010). Taxon-specific responses of soil bacteria to the addition of low level C inputs. Soil Biol. Biochem..

[76] Argiroff W.A., Zak D.R., Upchurch R.A., Salley S.O., Grandy A.S. (2019). Anthropogenic N deposition alters soil organic matter biochemistry and microbial communities on decaying fine roots. Glob. Chang. Biol..

[77] Eichorst S.A., Trojan D., Roux S., Herbold C.W., Rattei T., Woebken D. (2018). Genomic insights into the Acidobacteria reveal strategies for their success in terrestrial environments. Environ. Microbiol..

[78] Davis K.E.R., Sangwan P., Janssen P.H. (2011). Acidobacteria, Rubrobacteridae and Chloroflexi are abundant among very slow-growing and mini- colony-forming soil bacteria. Environ. Microbiol..

[79] Yan G., Xing Y., Xu L., Wang J., Dong X., Shan W., Guo L., Wang Q. (2017). Effects of different nitrogen additions on soil microbial communities in different seasons in a boreal forest. Ecosphere.

[80] Fierer N., Bradford M.A., Jackson R.B. (2007). Toward an ecological classification of soil bacteria. Ecology.

[81] Li C., Yan K., Tang L., Jia Z., Li Y. (2014). Change in deep soil microbial communities due to long-term fertilization. Soil Biol. Biochem..

[82] Sul W.J., Asuming-Brempong S., Wang Q., Tourlousse D.M., Penton C.R., Rodrigues J.L., Adiku S.G., Jones J.W., Zhou J. (2013). Tropical agricultural land management influences on soil microbial communities through its effect on soil organic carbon. Soil Biol. Biochem..

[83] Zhong Y., Yan W., Shangguan Z. (2015). Impact of long-term N additions upon coupling between soil microbial community structure and activity, and nutrient-use efficiencies. Soil Biol. Biochem..

[84] Feng Y., Grogan P., Caporaso J.G., Zhang H., Lin X., Knight R., Chu H. (2014). pH is a good predictor of the distribution of anoxygenic purple phototrophic bacteria in artic soils. Soil Biol. Biochem..

[85] Li P., Wu G., Li Y., Hu C., Ge L., Zheng X., Zhang J., Chen J., Zhang H., Bai N. (2022). Long-term rice-crayfish-turtle co-culture maintains high crop yields by improving soil health and increasing soil microbial community stability. Geoderma.

[86] Hu S., Xu C., Xie Y., Ma L., Niu Q., Han G., Huang J. (2024). Metagenomic insights into the diversity of 2,4-dichlorophenol degraders and the cooperation patterns in a bacterial consortium. Sci. Total Environ..

[87] Ke W., Li C., Zhu F., Luo X., Li X., Wu C., Hartley W., Xue S. (2023). The assembly process and co-occurrence patterns of soil microbial communities at a lead smelting site. Sci. Total Environ..

[88] de Vries F.T., Griffiths R.I., Bailey M., Craig H., Girlanda M., Gweon H.S., Hallin S., Kaisermann A., Keith A.M., Kretzschmar M. (2018). Soil bacterial networks are less stable under drought than fungal networks. Nat. Commun..

[89] Banerjee S., Schlaeppi K., van der Heijden M.G. (2018). keystone taxa as drivers of microbiome structure and functioning. Nat. Rev. Microbiol..

[90] Du M., Zhang J., Wang G., Liu C., Wang Z. (2022). Response of bacterial community composition and co-occurrence network to straw and straw biochar incorporation. Front. Microbiol..

[91] Chen J., Xiao Q., Xu D., Li Z., Chao L., Li X., Liu H., Wang P., Zheng Y., Liu X. (2023). Soil microbial community composition and co-occurrence network responses to mild and severe disturbances in volcanic areas. Sci. Total Environ..

[92] Zhang Y.D., Gao M., Yu C.Y., Zhang H.B., Yan N., Wu Q.M., Song Y.H., Li X.N. (2022). Soil nutrients, enzyme activities, and microbial communities differ phylum Proteobacteria among biocrust types and soil layers in a degraded karst ecosystem. Catena.

[93] Li R., Li Q., Zhang J., Liu Z., Pan L., Huang K., Zhang L. (2020). Effects of organic mulch on soil moisture and nutrients in karst area of southwest China. Pol. J. Environ. Stud..

[94] You J., Das A., Dolan E.M., Hu Z. (2009). Ammonia-oxidizing archaea involved in nitrogen removal. Water Res..

[95] Green P.N., Dworkin M., Falkow S., Rosenberg E., Schleifer K.-H., Stackebrandt E. (2006). Methylobacterium. The Prokaryotes: Volume 5: Proteobacteria.

