# Supplementary material for: Response of Soil Bacteria to Short-Term Nitrogen Addition in Nutrient-Poor Areas
Source: Microorganisms. 2025 Jan 1;13(1):56. doi: 10.3390/microorganisms13010056 (PMC11767588; doi:10.3390/microorganisms13010056)
Supplement: Supplementary file 1 [file microorganisms-13-00056-s001.zip › microorganisms-3323963-supplementary.pdf]

Table S1 Redundancy analysis explains the importance of variables and tests their significance

	Environmental factor	rdaenvfit		rda.hp		permu_hp	
		r2	p-value	Individual	I.perc(%)	Individual	Pr(>I)
phylum level	AK	0.846664	0.001	0.1773	23.8	0.1773	0.008 **
	pH	0.875323	0.001	0.0935	12.55	0.0935	0.029 *
	TOC	0.849252	0.001	0.1002	13.45	0.1002	0.025 *
	TN	0.84684	0.001	0.0921	12.36	0.0921	0.042 *
	TP	0.26513	0.014	0.023	3.09	0.023	0.216
	DOC	0.671149	0.001	0.0424	5.69	0.0424	0.127
	AP	0.792718	0.001	0.0466	6.26	0.0466	0.091
	NO_3	0.626889	0.001	0.0533	7.15	0.0533	0.089
	NH_4	0.392242	0.003	0.0182	2.44	0.0182	0.204
	Moisture	0.7456	0.001	0.0367	4.93	0.0367	0.137
	C:N	0.429288	0.002	0.0258	3.46	0.0258	0.186
	DON	0.532535	0.001	0.0358	4.81	0.0358	0.149
genus level	AK	0.877907	0.001	0.1746	24.77	0.1746	0.004**
	pH	0.844682	0.001	0.0714	10.13	0.0714	0.032*
	TOC	0.958322	0.001	0.0751	10.65	0.0751	0.038*
	TN	0.934415	0.001	0.0679	9.63	0.0679	0.047*
	TP	0.19882	0.05	0.0137	1.94	0.0137	0.232
	DOC	0.748805	0.001	0.044	6.24	0.044	0.092
	AP	0.751232	0.001	0.0454	6.44	0.0454	0.082
	NO_3	0.902582	0.001	0.0534	7.57	0.0534	0.06
	NH_4	0.870231	0.001	0.0462	6.55	0.0462	0.09

	Moisture	0.686633	0.001	0.049	6.95	0.049	0.091
	C:N	0.159034	0.107	0.0207	2.94	0.0207	0.173
	DON	0.572326	0.001	0.0432	6.13	0.0432	0.095

Lai Jiangshan, Zou Yi, Zhang Jinlong, Peres-Neto Pedro (2022). Generalizing hierarchical and variation partitioning in multiple regression and canonical analyses using the rdacca.hp R package. Methods in Ecology and Evolution, 13: 782-788<DOI:10.1111/2041-210X.13800>

Table S2 Topological properties of co-occurrence network obtained from different nitrogen additional treatments

	CK	N1	N2	N3	N4
average degree	29.709	16.658	16.695	22.304	23.358
network diameter	15.019	18.763	14.051	19.749	14.093
network density	0.083	0.044	0.045	0.062	0.063
modularity	0.505	0.609	0.86	0.548	0.511
average clustering coefficient	0.572	0.544	0.575	0.582	0.575
average path length	3.357	4.984	4.776	4.543	4.363

Table S3 Physicochemical factors and microbial biomass of soils sampled from different nitrogen additional treatments

Nitrogen treatment	CK	2g	4g	8g	16g
TC g kg^{-1}	9.922±0.024d	11.420±0.301cd	12.223±0.595c	24.102±0.485a	14.065±0.898b
TN g kg^{-1}	0.672±0.021c	0.748±0.033c	0.670±0.016c	1.322±0.030a	0.972±0.056b
TP g kg^{-1}	2.105±0.026a	2.035±0.029a	1.793±0.022c	1.862±0.022c	1.955±0.031b
DOC mg kg^{-1}	38.70±3.08c	42.16±1.42c	53.43±3.06b	95.35±1.89a	52.47±1.38b
DON mg kg^{-1}	15.03±0.45d	18.31±0.23c	12.79±0.49e	29.90±0.15b	57.94±0.74a
NH_4-N mg kg^{-1}	3.53±0.09e	4.42±0.05d	7.83±0.13c	15.92±0.22b	48.73±0.61a
NO_3-N mg kg^{-1}	6.40±0.08d	8.65±0.08c	2.76±0.21e	13.91±0.23b	20.29±0.50a
SAP mg kg^{-1}	0.78±0.01d	1.25±0.02c	1.72±0.03c	7.20±0.16b	10.65±0.07a
AK mg kg^{-1}	207.29±4.76d	304.94±8.11c	302.39±10.85c	361.22±15.92b	407.85±9.42a
SM	0.182±0.004c	0.185±0.004c	0.186±0.003c	0.245±0.004a	0.211±0.005b
pH	6.69±0.078a	6.79±0.079a	6.71±0.080a	6.20±0.073b	5.83±0.087c
MBC mg kg^{-1}	234.21±13.76c	290.86±7.83b	315.65±3.29b	485.01±12.20a	155.78±4.67d
MBN mg kg^{-1}	25.18±0.25c	32.51±1.12b	32.81±1.49b	56.55±1.52a	35.14±1.85b
MBP mg kg^{-1}	4.27±0.44cd	4.54±0.07c	3.63±0.10d	15.44±0.33a	5.64±0.13b

Note: TC: total carbon concentration, TN: total nitrogen concentration, TP: total phosphorus concentration, DOC: dissolved organic carbon, DON: dissolved organic nitrogen, NH_4-N: ammonium nitrogen concentration, NO_3-N: nitrate nitrogen concentration, SAP: soil available phosphorus concentration, AK: soil available potassium concentration, pH: soil pH, SM: soil moisture content, MBC: microbial biomass carbon, MBN: microbial biomass nitrogen, MBP: microbial biomass phosphorus.

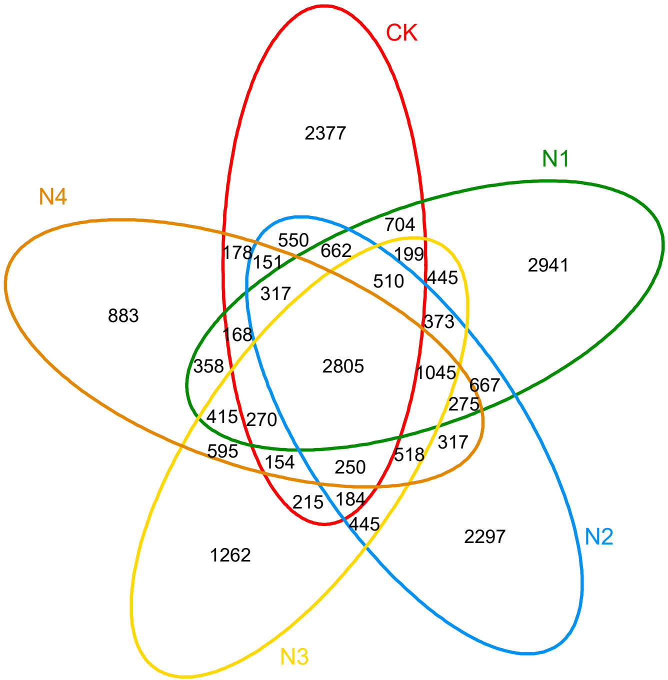

Fig. S1 Venn to show the cluster distribution of the bacteria at different nitrogen

addition treatments on OTU level

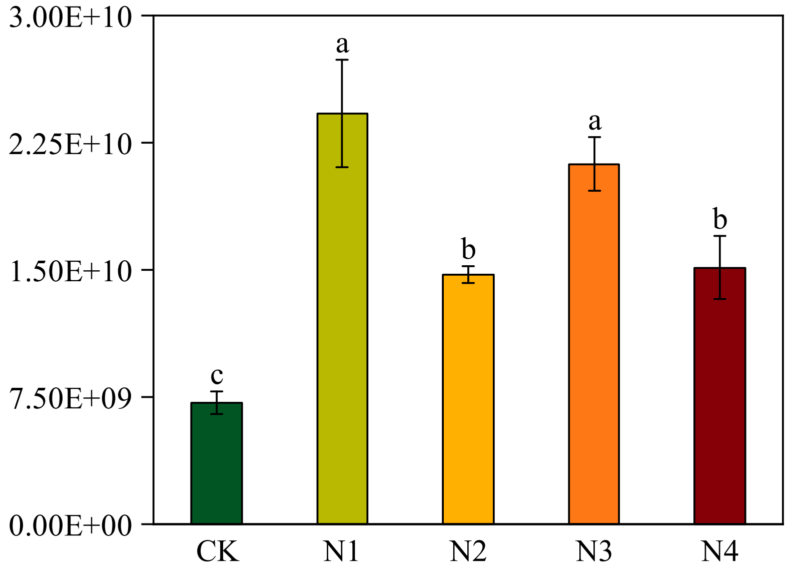

Fig. S2 The variation characteristics of 16S gene copy number at different nitrogen addition treatments

Different lowercase letters indicate significant differences of 16S gene copy number between different nitrogen addition treatments ($P < 0.05$).

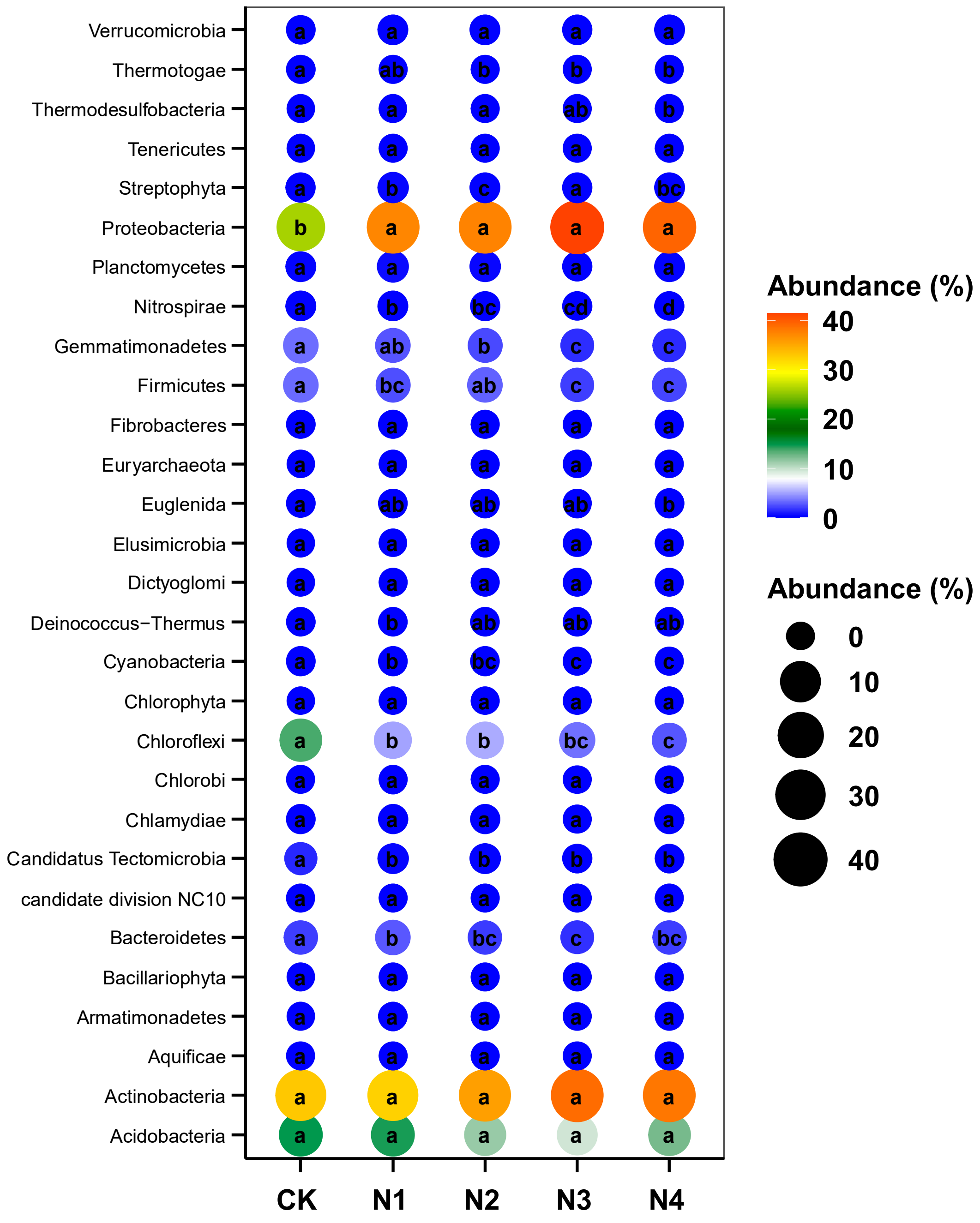

Figure S3. Significance test of bacterial community composition at different nitrogen addition treatments on phylum level

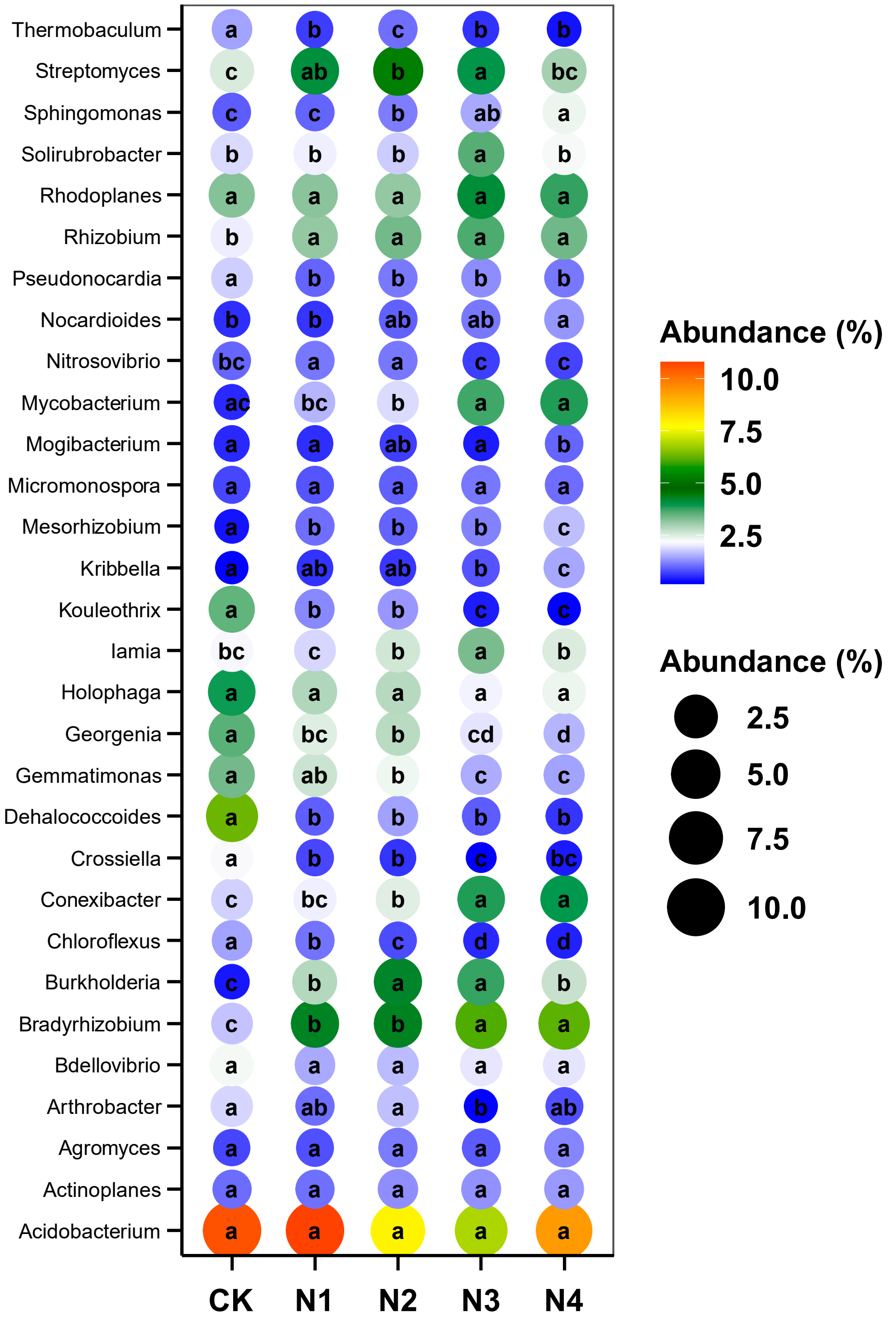

Figure S4. Significance test of bacterial community composition at different nitrogen addition treatments on genus level

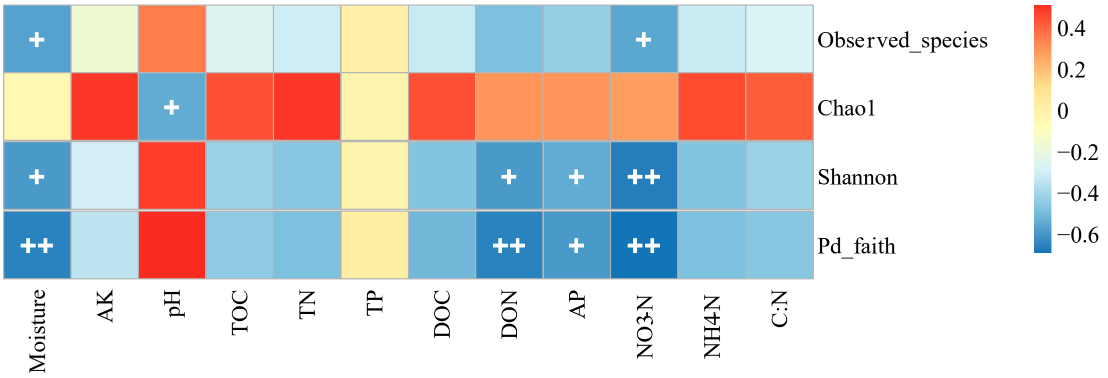

Figure S5. Heatmap of the correlation between bacterial alpha diversity and environmental factors

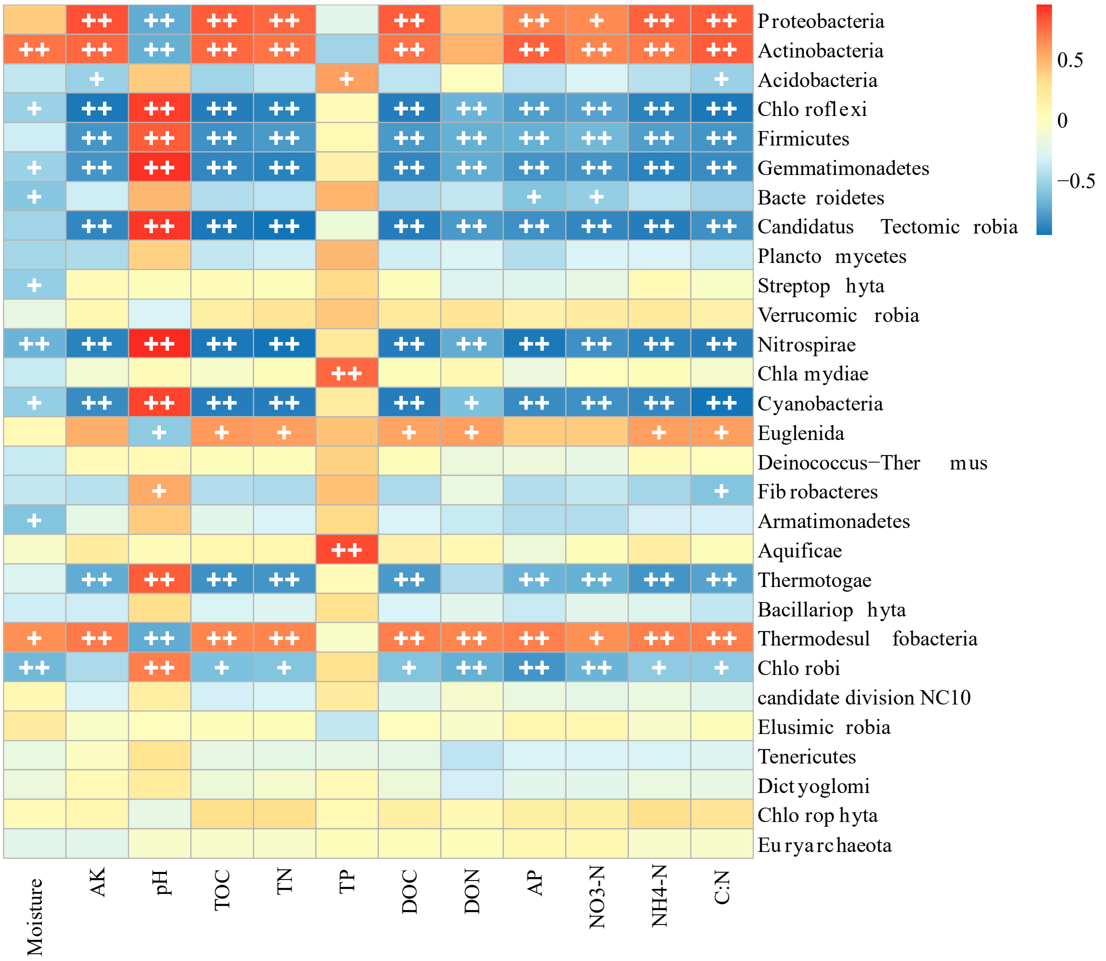

Figure S6. Heatmap of the correlation between nitrogen addition treatments and environmental factors on phylum level

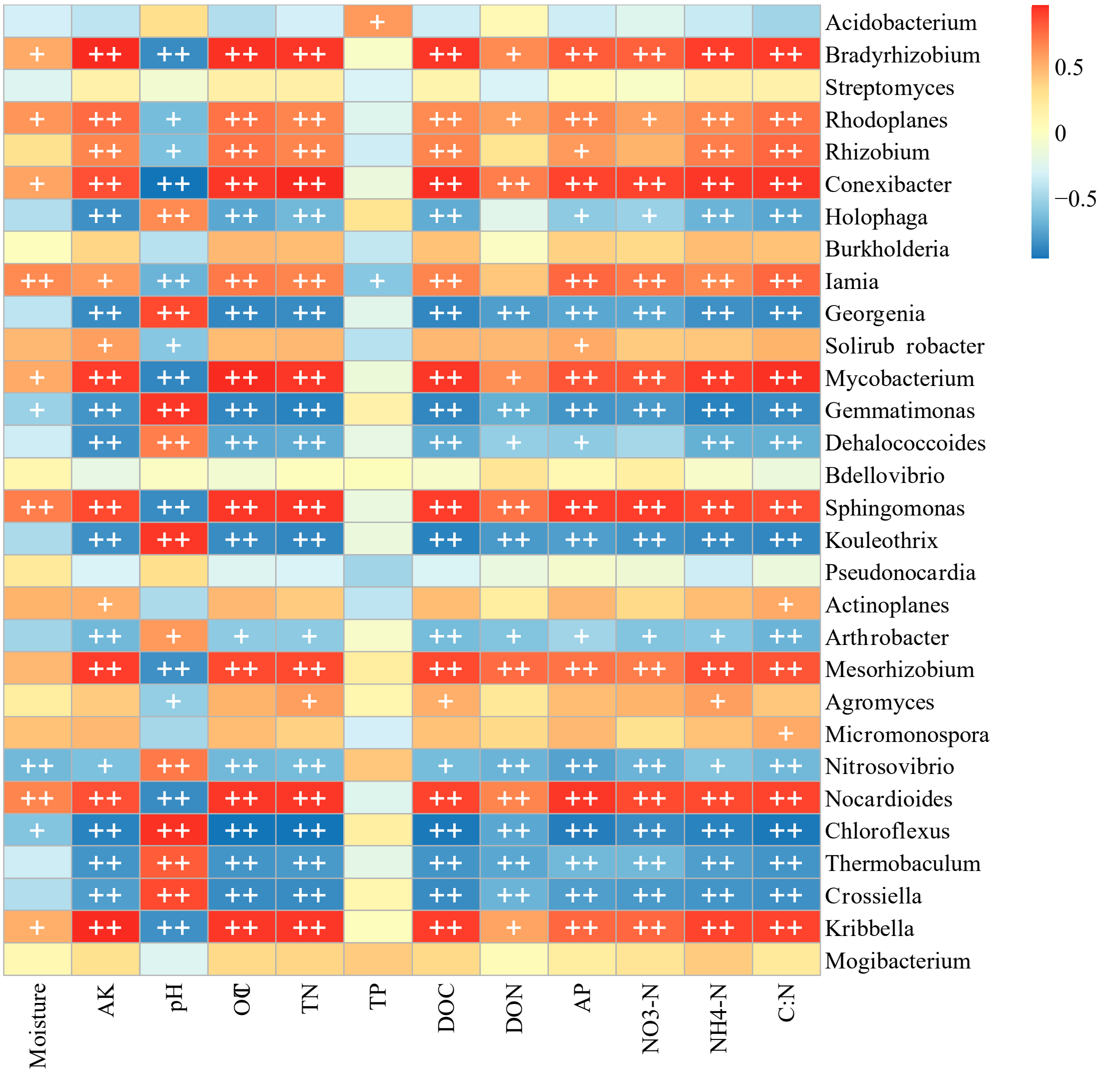

Figure S7. Heatmap of the correlation between nitrogen addition treatments and environmental factors on genus level

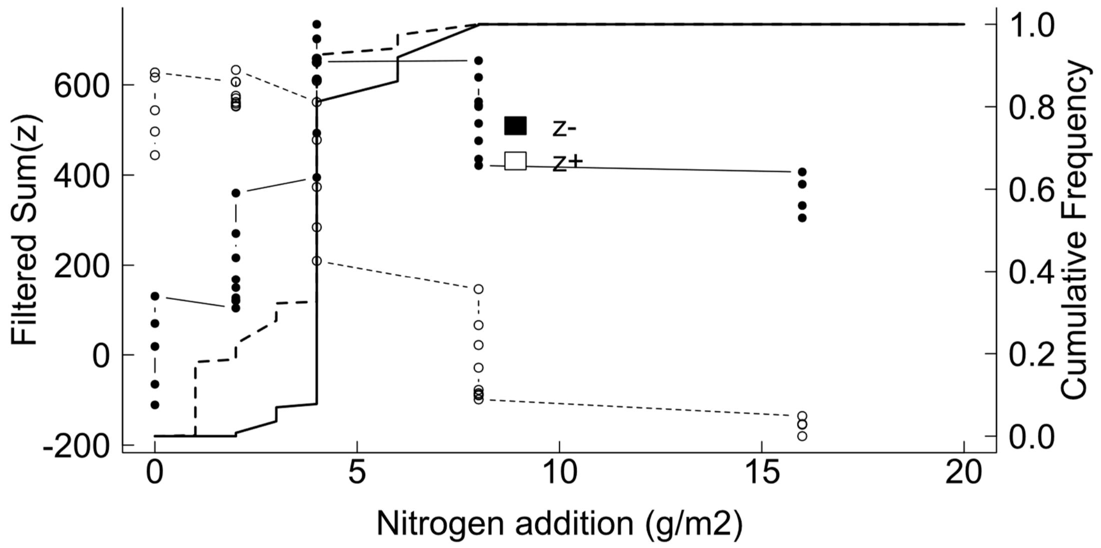

Figure S8. Bacterial community change point for genus reduced in abundance along with N addition levels showing community threshold at maximum [sum(z)] and 5-95% bootstrap percentile range. The sum(z) values represent the sum of responses for each possible change point along with N addition levels. The labels marked inside the x-axis represent the specific N addition levels in this study.
